# Factors Influencing Social Participation Among Persons With Neuromyelitis Optica Spectrum Disorders: A Cross-Sectional Study

**DOI:** 10.3389/fneur.2022.843909

**Published:** 2022-05-09

**Authors:** Mengmeng Jia, Ping Fan, Lin Ning, Hengfang Ruan, Tong Wang, Wei Qiu, Huijuan Li, Kun Li

**Affiliations:** ^1^School of Nursing, Sun Yat-sen University, Guangzhou, China; ^2^Department of Neurology, The Third Affiliated Hospital of Sun Yat-sen University, Guangzhou, China; ^3^Department of Sleep Medicine and Department of Cardiopulmonary Rehabilitation, The First Affiliated Hospital of Guangzhou University of Chinese Medicine, Guangzhou, China

**Keywords:** neuromyelitis optica, social participation, activities of daily living, anxiety, depression

## Abstract

**Background:**

Neuromyelitis optica spectrum disorders seriously hinder social participation. Only clarifying the status of social participation and the factors most strongly influencing it can promote better-targeted support for those with neuromyelitis optica spectrum disorders.

**Objectives:**

To explore the factors influencing social participation among persons with neuromyelitis optica spectrum disorders.

**Methods:**

138 persons with neuromyelitis optica were recruited by convenience sampling. They completed the Impact on Participation and Autonomy Questionnaire, and were rated using the Barthel index, the Beck Anxiety Inventory and the Beck Depression Inventory. The data were used to evaluate a generalized linear model predicting social participation.

**Results:**

The respondents mostly perceived good or very good possibilities of social participation, but participation outdoors and in social relations were considered more restricted. Ability in the activities of daily living, education level, bladder and bowel dysfunction, and depression were four significant predictors of social participation.

**Conclusions:**

Strengthening training in the activities of daily living, continuing education, giving positive psychological support and helping with bladder and bowel problems may help to promote social participation among those with neuromyelitis optica spectrum disorders.

## Introduction

Neuromyelitis optica spectrum disorders (NMOSD) are a group of antigen-antibody-mediated inflammatory disorders of the central nervous system mainly involving humoral immunity (1, 2), including neuromyelitis optica (NMO) and some diseases which have pathogenesis and clinical features similar to those of NMO. Prior to 2004, NMO was often regarded as a specific subtype of multiple sclerosis (MS), but the recent discovery of AQP4 and NMO-IgG supports considering it as an independent disease (3–5). A large cohort study (6) has reported NMOSD to be the most common inflammatory demyelinating disease of the central nervous system in China. It is highly recurrent and very disabling, and has a high incidence among young and middle-aged people, especially women. More than 90% of patients report single or multiple relapses within 3 years. Most patients present with irreversible damage to the optic nerve and spinal cord manifested as severe visual impairment, limb dysfunction, bladder and bowel disorders, and eventually paralysis (7–10). Although there has been much progress in NMOSD maintenance therapy since 2019 (11–13), there are only a few treatments which are generally recognized, and the high costs of long-term treatment and medication burden NMOSD patients (14). Along with economic pressure, negative emotions, social phobia and work limitations seriously hinder patients' return to their families and to society (15).

Social participation is usually closely related to the quality of life and is considered an important determinant of health status (16). Only by clarifying social participation status and the factors that influence it will it be possible to better promote social reintegration in a more targeted manner. However, previous studies of NMOSD have mostly focused on pathology, mechanisms and treatment. Social participation has rarely been a research focus. This despite the fact that the work which has been done indicates that 63% of patients rarely or never participate in social activities due to their illness, and 60% are unable to continue working (15). Apart from that, there are almost no specific data on other aspects of social participation by persons with NMOSD, especially in China.

The best predictors of social participation among persons with NMOSD are also not clear. But since NMOSD and MS are both central nervous system inflammatory diseases, the results of research among patients with MS may be generalizable to the NMOSD population. Those results show that patients' social participation correlates with functional ability, negative emotions such as depression and anxiety (17–19) and other demographic and disease-related characteristics, including the course of the disease, income and so on (17). NMOSD entails severe dysfunction including visual impairment, lower limb paralysis, urinary retention and sensory impairment, and its activity limitations are usually more severe than among persons with MS. So a high incidence of negative emotions such as depression and anxiety should not be surprising (20, 21).

This study was therefore designed to explore the status of persons with NMOSD and the factors influencing their social participation. The objective was to provide a theoretical basis for better targeting measures to promote such persons' social participation.

## Methods

### Participants

This was a cross-sectional study conducted in the Third Affiliated Hospital of Sun Yat-sen University in Guangzhou, China. Its protocols were approved by the hospital's Medical Ethics Committee ([2021]02-010-01).

Discharged patients who met the diagnostic criteria for NMOSD (1) were recruited in April 2021 by convenience sampling from the hospital's follow-up system. Patients hospitalized for acute recurrence or under the age of 18 were excluded. The candidates were contacted by telephone to obtain verbal informed consent. They were then sent online questionnaires which the participants completed independently. They could not be submitted until all of the items had been completed. A minimum necessary sample size of 90 was estimated using regression analysis (22).

### Measurements

#### General Information

The demographic information solicited included gender, age, level of education, employment status, and the family's per capita monthly income. Information was also solicited about the course of the disease, any co-morbidity, recurrence incidence, medications, AQP4 test results and major obvious symptoms such as impaired vision, bladder and bowel dysfunction, motor deficits, digestive symptoms, pain, abnormal skin sensation and impaired balance.

#### Neurological Status

The expanded disability scale (EDSS) developed by Kurtzke in 1983 is the physical disability assessment scale most widely used with NMOSD patients (23). The EDSS evaluates eight functional abilities related to the central nervous system. It deals with impaired sensation (such as visual impairment and numbness) and also motor limitations (activity, walking, etc.). The scores range from 0 to 10, with higher scores indicating greater disability.

#### Social Participation

The Impact on Participation and Autonomy Questionnaire (IPA) was administered to assess the participants self-perceived needs in terms of autonomy and participation (24). That instrument has shown good reliability and validity (25). The revised Chinese version solicits responses on 25 items using 5-point Likert scales. Its four dimensions are autonomy indoors, family role, autonomy outdoors and social relations. Participants were asked to select from “very large,” “large,” “average,” “small,” or “very small” to describe their self-perceived possibility of participating in social life. The scores then ranged from 0 to 4. The higher the score, the worse the prospects of social participation.

#### Activities of Daily Living

The Barthel index (BI) is widely used to quantify ability in the activities of daily living (ADL). It is well-known to offer high reliability and simple evaluation (26). It has 10 items scored “0,” “5,” “10,” or “15” to indicate ADL ability. The total possible score is 100, with 0–40 indicating heavy dependence, 41–60 moderate dependence, 61–99 mild dependence and 100 complete independence.

#### Anxiety

The Beck Anxiety Inventory (BAI) was used to evaluate any somatic symptoms of anxiety, including nervousness, dizziness, inability to relax and others (27). It was translated into Chinese in 2001 and has shown good reliability and validity with Cronbach's α of 0.95 (28). The BAI has 21 items, each scored as 0 (not at all) to 3 (severely). The total score can range from 0 to 63, with 0–9 considered normal with no undue anxiety, 10–18 indicating mild anxiety, 19–29 moderate anxiety, and 30–63 severe anxiety.

#### Depression

The Beck Depression Inventory (BDI-I) is one of the earliest and most commonly-used self-rating scales for depressive symptoms in Chinese populations. Version 1 has been confirmed to have good reliability and validity (29, 30). The BDI-I consists of 21 items. The participants rated themselves on a scale of 0 to 3 according to any depressive symptoms they experienced in the previous week. Here too the total score can range from 0 to 63, with a score between 0 and 13 indicating no depression, 14–19 indicating mild depression, 20–28 moderate depression, and 29–63 severe depression.

### Statistical Analysis

Version 25.0 of the SPSS software suite (IBM, Armonk, NY) was used for the statistical analyses. Distributions were computed for the participants' general information items. Their IPA, BI, BAI and BDI scores were described in terms of frequency, percentages, medians and inter-quartile ranges. Non-parametric tests and Spearman rank correlation analysis were conducted to test the significance of differences in and relationships among the social participation and other variables. And for the multiple comparisons of IPA scores among different levels of polytomous variables, the *p*-values were adjusted with the Bonferroni correction. All of the variables which proved to be significant in the univariate analysis were incorporated into generalized linear models exploring the factors influencing social participation.

## Results

### Demographics

There were 138 persons with NMOSD who completed the questionnaire online. 95.7% were female, and their ages were mostly concentrated in the 18–44 years range. More than half of the respondents admitted to being out of work, and most claimed a family per capita monthly income between ¥1,000 and 5,000. 84.1% had obvious symptoms. The median of course of disease was 5 years with an average recurrence of twice. Almost all of the respondents said they used a glucocorticoid, an immunosuppressant and/or some other medication. 93.2% of those who had taken the AQP4 test by the indirect immunofluorescence method had tested positive (Table 1).

**Table 1:** Characteristics of the respondents and the results of univariate analyses (*N* = 138).

**Variable**	***n* (%) or median (Q_**1**_, Q_**3**_)^a^**	***U/H/r_s_* ^b^**	***p* ^c^**
**Gender**		−0.372	0.710
Male	6 (4.3)		
Female	132 (95.7)		
**Age (year)**	38 (32, 51)	12.026	0.002
18–44①	89 (64.5)		0.022
45–59②	39 (28.3)		0.025
60–71③	10 (7.2)		0.914
**Education level**		17.932	≤ 0.001
Primary school or below①	15 (10.9)		0.159
Secondary school②	67 (48.6)		0.000
Higher education③	56 (40.6)		0.007
**Employment**		4.728	≤ 0.001
Yes	55 (39.9)		
No	83 (60.1)		
**Family per capita monthly income (yuan)**		8.352	0.015
<1,000①	15 (10.9)		0.410
1,000–5,000②	81 (58.7)		0.021
>5,000③	42 (30.4)		0.116
**EDSS score (*****n*** **=** **75)^#^**	3 (2, 3.5)	0.337	0.003
0–4.5	66 (88.0)		
5–7.5	7 (9.3)		
>7.5	2 (2.7)		
**AQP4 results (*****n*** **=** **74)^#^**		0.679	0.516
Positive	69 (93.2)		
Negative	5 (6.8)		
**Course of disease (year)**	5 (3,10)	0.061	0.476
**Co-morbidity**		3.049	0.002
Yes	55 (39.9)		
No	83 (60.1)		
**Recurrence times (** ***n*** **=133)** ^*^	2 (1,5)	0.180	0.038
**Obvious symptoms**		−4.422	≤ 0.001
Yes	116 (84.1)		
Impaired vision	81 (58.7)	1.411	0.158
Bladder and bowel dysfunction	31 (22.5)	3.614	≤ 0.001
Motor deficits	45 (32.6)	3.969	≤ 0.001
Digestive symptoms	10 (7.2)	1.124	0.261
Pain	41 (29.7)	1.831	0.067
Abnormal skin sensation	27 (19.6)	0.027	0.979
Balance disorder	19 (13.8)	2.779	0.005
No	22 (15.9)		
**Medications**		–	–
Glucocorticoids	110 (79.7)		
Immunosuppressant	119 (86.2)		
Others (vitamin, acesodyne)	47 (34.1)		
Not clear	5 (3.6)		
**IPA score^d^**	0.50 (0.14, 1.27)	–	–
Autonomy indoors	0 (0, 0.46)		
Family role	0.29 (0, 1.29)		
Autonomy outdoors	1.00 (0.20, 1.85)		
Social relations	0.67 (0.17, 1.33)		
**BI score**	100 (95, 100)	−0.487	≤ 0.001
Independence	93 (67.4)		
Mild dependence	39 (28.3)		
Moderate dependence	3 (2.2)		
Heavy dependence	3 (2.2)		
**BDI**-I **score**	14 (7, 22.25)	0.597	≤ 0.001
No depression	68 (49.3)		
Mild	22 (15.9)		
Moderate	27 (19.6)		
Severe	21 (15.2)		
**BAI score**	32 (26, 39.25)	0.485	≤ 0.001
Moderate	58 (42.0)		
Severe	80 (58.0)		

*EDSS, expanded disability status scale; IPA, Impact on Participation and Autonomy Questionaire; BI, Barthel index; BAI, Beck Anxiety Inventory; BDI-I, Beck Depression Inventory version 1*.

a *Variables which were not normally distributed are described by their medians and lower and upper quartiles (Q1, Q3)*.

b *U for the Mann-Whitney non-parametic U-test; H for the Kruskal-Wallis test; r_s_ for Spearman rank correlation analysis*.

c *The p-values of univariate analyses between IPA scores and other variables. For multiple comparisons about age, education level and family per capita monthly income, the p-values have been adjusted by the Bonferroni correction*.

d *The average score on the items*.

# *The missing data were from patients who were admitted for relapses and did not do the tests again*.

* *There were five patients who couldn't recall a specific number of recurrences*.

The non-parametric tests revealed significant differences in the average IPA scores among different groups by age (*H* = 12.026, *p* ≤ 0.01), education level (*H* = 17.932, *p* ≤ 0.001), employment (*U* = 4.728, *p* ≤ 0.001), family per capita monthly income (*H* = 8.352, *p* ≤ 0.05), and between those with and without co-morbidity (*U* = 3.049, *p* ≤ 0.01) or obvious symptoms (*U* = −4.422, *p* ≤ 0.001). In addition, the IPA scores were significant higher in groups with bladder and bowel dysfunction (*U* = 3.614, *p* ≤ 0.001), motor deficits (*U* = 3.969, *p* ≤ 0.001) or impaired balance (*U* = 2.779, *p* ≤ 0.01). Spearman rank correlation analysis also revealed significant correlation between social participation and the number of times NMOSD had recurred (*r*_s_ = 0.180, *p* ≤ 0.05).

### Social Participation

The median IPA item score was 0.50 with an interquartile range of (0.14, 1.27). Most respondents considered their prospects of participating in social life as good or very good. They considered their autonomy outdoors [1.00 (0.20, 1.85)] the greatest impediment, followed by limited social relations [0.67 (0.17, 1.33)] and family role limitations [0.29 (0, 1.29)], with autonomy indoors [0 (0, 0.46)] causing the least difficulty (Table 1).

### Activities of Daily Living, Neurological Status, Anxiety and Depression

The average BI score was 100, indicating only mild dependence overall. 32.6% of the respondents reported some degree of self-care problems, among whom six considered themselves moderately or heavily dependent. As for neurological status, among the 75 who could report an EDSS score, 88.0% had scores in the 0–4.5 range, indicating no walking limitations. However, the score of two respondents was more than 7.5, indicating that they could barely walk. The median BAI score was 32. Everyone reported moderate or severe anxiety. Despite that, the average level of depression was mild [14 (7, 22.25)], though nearly half of the respondents reported some level of depression.

The Spearman rank correlation analysis showed a negative correlation between the IPA and BI scores (*r*_s_ = −0.487, *p* ≤ 0.001), and that the IPA scores were positively correlated with the EDSS scores (*r*_s_ = 0.337, *p* ≤ 0.01), the BAI scores (*r*_s_ = 0.485, *p* ≤ 0.001) and the BDI-I scores (*r*_s_ = 0.597, *p* ≤ 0.001) (Table 1).

### Factors Influencing Social Participation

The first generalized linear model based on full data from 71 respondents showed no significant relationship between EDSS scores and social participation. That predictor was therefore removed in the following models.

The final model used data from 133 respondents. Its coefficients are shown in Table 2. An omnibus test showed that the model had useful predictive power (χ^2^ = 131.518, *p* ≤ 0.001). Because higher IPA score indicate less social participation, ADL ability was a positive predictor of social participation (β = −0.024, *p* ≤ 0.001) and depression (β = 0.031, *p* ≤ 0.001) and bladder and bowel dysfunction (β = −0.249, *p* ≤ 0.05) were negative predictors. Education level was also a positive predictor (β = 0.507, *p* ≤ 0.001).

**Table 2:** Coefficients and statistics of the final generalized linear model of factors influencing social participation^a^ (*N* = 133).

**Variable**	**β**	**S-_***x***_**	***Wald* ^′^ *sχ* ^2^**	***p***	**OR (95% CI)**
Constant	2.676	0.4192	40.767	≤ 0.001	14.532 (6.390–33.047)
**Education level**					
Primary school or below	0.507	0.1585	10.242	≤ 0.001	1.661 (1.217–2.266)
Secondary school	0.123	0.0973	1.587	0.208	1.130 (0.934–1.368)
Higher education	0^b^				
**Bladder and bowel dysfunction**					
No	−0.249	0.1142	4.747	0.029	0.780 (0.623–0.975)
Yes	0^b^				
**BI**	−0.024	0.0041	33.870	≤ 0.001	0.976 (0.968–0.984)
**BDI**-I	0.031	0.0047	43.183	≤ 0.001	1.032 (1.022–1.041)

*OR, Odds ratio; CI, confidence interval; BI, Barthel index; BDI-I, Beck Depression Inventory version 1*.

a *Results of an Omnibus test: χ^2^ = 131.518, p ≤ 0.001*.

b *Reference*.

## Discussion

The study's respondents with NMOSD generally perceived some possibility of social participation. They felt more restrictions on their autonomy outdoors and in social relations than indoors and in family roles. Their social participation was related to their ADL ability, level of education, bladder and bowel dysfunction and any feelings of depression.

This study's participants generally perceived a high likelihood of their participating in society, which differs sharply from the findings of previous studies of persons with MS (17, 19). Indeed, it was rather unexpected. Similar research among persons with MS produced an average IPA item score of 2.05. They perceived little possibility of social participation on average (17). The average IPA item score in this study was 0.5, reflecting a strong or very strong likelihood of social participation. That reflects the low overall level of dysfunction among the respondents. Most of them had EDSS scores in the 0–4.5 range, with no limitation on their walking ability and a low level of dysfunction overall (31). The results of the univariate analyses showed that the respondents' social participation was negatively correlated with their EDSS scores, confirming that less dysfunction allows better social participation. This suggests that future studies should pay more attention to patients with poor functional abilities and that programs to help them improve their social participation should be improved.

That autonomous social participation outdoors and in social relations was more restricted than that indoors and in family roles is not unexpected. The disease does affect social participation to a certain extent, especially in terms of outdoor activities and socializing. This may be mainly related to the inconvenience of traveling with NMOSD. Compared with the familiar and convenient family environment, unfamiliar environments and dealing with any defecation problems when going out may be seen as major deterrents (32, 33). And this study has also confirmed that bladder or bowel dysfunction is a negative predictor of social participation, as Mutch has previously reported (34). In addition, barrier-free facilities in China generally remain deficient (35). That may also discourage travel and social interaction. Previous studies have shown that NMOSD severely affects one's establishing social relationships, especially at work and in social activities. Most participate rarely or never in social activities, and they often are unable to go out to work. There might also be misunderstanding, discrimination and exclusion (15). Help with functional exercise, vocational training, and especially toilet training, providing barrier-free facilities and sufficient social support may be the keys to encouraging those with NMOSD to leave home and integrate into society.

One of factors found to predict social participation was ADL ability. Better ADL ability predicts better social participation. This has also been observed with other sorts of disability. A group led by Margaret, for example, found that persons with a spinal cord injury but good ADL ability also had high levels of social participation (36). Impaired ADL ability will not only will limit social activity, but also tend to generate negative emotions (37), which can further limit social participation. Therefore, ADL training may be an effective measure to promote social participation. It should be strengthened.

An interesting finding was that more education predicts more social participation among those with NMOSD. This may be because better-educated patients have a better understanding of their disease. They may also have more choices in their social activities than those with less education (38). Encouraging those with NMOSD to continue their education may have a positive effect on their social participation.

As would be expected, depression had a negative relationship with social participation. This is consistent with the findings about the participation of other disabled people (39). Depression is a negative emotional reaction, often manifested as persistent upset, even grief (40). Ayzenberg's group found that more than one-third of persons with NMOSD (39.8%) suffered from depression, with 51.5% of the cases moderate to severe (41). In this study 50.7% of the respondents reported various degrees of depressive symptoms. However, a group led by Motl has shown that effective participation can reduce the occurrence of depressive symptoms (42). Therefore, paying attention to a depression and its symptoms, and seeking effective relief measures, may create a virtuous circle, improving social participation and even overall quality of life.

It should be borne in mind that some of the patients included in this study had been hospitalized in the research center for disease recurrence. In some cases some disease data were missing and could not be obtained due to poor communication in China's hospital information systems. Type of abnormality and AQP4 and EDSS test results were the least reliably available. This problem will gradually be corrected. Note too that the overall level of dysfunction among the respondents was low, which would have influenced the responses. Although this study took into account some obvious symptoms of NMOSD and examined their ability to predict social participation, the role of fatigue was still overlooked (43). The limitations and obstacles to social participation for less able persons need further attention.

## Conclusions

Persons with NMOSD have good prospects for social participation overall, but feel significantly restricted in outdoor activities and in socializing. ADL ability, bladder and bowel dysfunction, depression and education level are the significant predictors of social participation. Healthcare staff and caregivers can promote social participation by strengthening ADL training, helping with bladder and bowel problems, giving positive psychological support, encouraging patients to continue their education and improving their understanding of NMOSD.

## Data Availability Statement

The original contributions presented in the study are included in the article, further inquiries can be directed to the corresponding authors.

## Ethics Statement

The studies involving human participants were reviewed and approved by the Medical Ethics Committee of the Third Affiliated Hospital of Sun Yat-sen University ([2021]02-010-01). Written informed consent from the patients/participants or patients/participants' legal guardian/next of kin was not required to participate in this study in accordance with the national legislation and the institutional requirements.

## Author Contributions

MJ and KL were responsible for the statistical analysis and drafted and revised the manuscript. PF, LN, HR, TW, WQ, and HL were responsible for the study's design, data collection, and data management. All of the authors agree to be accountable for the content of the work. All authors contributed to the article and approved the submitted version.

## Funding

This work was supported by China's National Natural Science Foundation (grant 72004240), the Special Cultivation Funding of the Third Affiliated Hospital of Sun Yat-sen University (grant 2020GZRPYQN12), and as a Natural Science Foundation Project of Guangdong Province's Basic and Applied Basic Research Foundation (no. 2021A1515011800).

## Conflict of Interest

The authors declare that the research was conducted in the absence of any commercial or financial relationships that could be construed as a potential conflict of interest.

## Publisher's Note

All claims expressed in this article are solely those of the authors and do not necessarily represent those of their affiliated organizations, or those of the publisher, the editors and the reviewers. Any product that may be evaluated in this article, or claim that may be made by its manufacturer, is not guaranteed or endorsed by the publisher.
